# A New *Z*-axis Resonant Micro-Accelerometer Based on Electrostatic Stiffness

**DOI:** 10.3390/s150100687

**Published:** 2015-01-05

**Authors:** Bo Yang, Xingjun Wang, Bo Dai, Xiaojun Liu

**Affiliations:** 1 School of Instrument Science & Engineering, Southeast University, Nanjing 210096, China; E-Mails: wangxingjun2000@126.com (X.W.); cumtdaibo@126.com (B.D.); liuxiaojun0814@126.com (X.L.); 2 Key Laboratory of Micro-Inertial Instrument and Advanced Navigation Technology, Ministry of Education, Nanjing 210096, China; 3 School of Information and Control, Nanjing University of Information Science & Technology, Nanjing 210044, China

**Keywords:** resonant micro-accelerometer, electrostatic stiffness, torsional accelerometer, plane resonator

## Abstract

Presented in the paper is the design, the simulation, the fabrication and the experiment of a new *z*-axis resonant accelerometer based on the electrostatic stiffness. The new *z*-axis resonant micro-accelerometer, which consists of a torsional accelerometer and two plane resonators, decouples the sensing movement of the accelerometer from the oscillation of the plane resonators by electrostatic stiffness, which will improve the performance. The new structure and the sensitive theory of the acceleration are illuminated, and the equation of the scale factor is deduced under ideal conditions firstly. The Ansys simulation is implemented to verify the basic principle of the torsional accelerometer and the plane resonator individually. The structure simulation results prove that the effective frequency of the torsional accelerometer and the plane resonator are 0.66 kHz and 13.3 kHz, respectively. Then, the new structure is fabricated by the standard three-mask deep dry silicon on glass (DDSOG) process and encapsulated by parallel seam welding. Finally, the detecting and control circuits are designed to achieve the closed-loop self-oscillation, to trace the natural frequency of resonator and to measure the system frequency. Experimental results show that the new *z*-axis resonant accelerometer has a scale factor of 31.65 Hz/g, a bias stability of 727 μg and a dynamic range of over 10 g, which proves that the new *z*-axis resonant micro-accelerometer is practicable.

## 1. Introduction

Resonant micro-accelerometers, which measure external acceleration through the frequency variation of a resonator, have good properties, such as the large dynamic range, the high sensitivity, the strong anti-interference ability, as well as the direct digital output. Therefore, a variety of resonant micro-accelerometers have been developed [1–4]. Due to the merits of the good linearity, the high accuracy and the process compatible with conventional silicon micromachining technology in the in-plane linear resonators, most of the resonant micro-accelerometers studied are currently used to measure the plane acceleration [4–9]. Only a few resonant accelerometers with out-of-plane resonators that can measure the acceleration in the vertical plane are investigated [10–14]. A resonant accelerometer with a single *z*-axis resonator is researched in the literature [10,11]. However, the natural frequency of the single resonator is extremely sensitive to the temperature variation, which will result in a spurious signal output. At the same time, it is difficult to ensure the stability of the resonant mode due to the sensing direction coinciding with the oscillation direction. Furthermore, the out-of-plane resonators have a large nonlinearity and are susceptible to the influence of the electrostatic pull-in effect. An alternative proposal [12] makes use of a tilting proof mass and two torsional resonators. Nevertheless, the sensing movement is still coupled with the oscillation of torsional resonators. In addition, resonant accelerometers based on the electromagnetic excitation have a poor process compatibility and bulky volume [13]. A vertical resonant accelerometer based on a nanoelectromechanical oscillator is beneficially attempted in the literature [14]. The work in [15] presents a new resonant silicon accelerometer based on differential frequency modulation. The in-plane accelerometer is transformed into frequency variation by electrostatic stiffness.

This paper focuses on the design of a new *z*-axis resonant micro-accelerometer, where the vertical accelerometer is converted into frequency variation by electrostatic stiffness. The new *z*-axis resonant micro-accelerometer, which consists of a torsional accelerometer and two plane resonators, decouples the sensing movement of the accelerometer from the oscillation of the plane resonators by electrostatic stiffness. In Section 2, the structure principle, the simulation and the fabrication are described briefly. In Section 3, the design of the oscillation loop and the frequency measurement circuit are presented. Then, the experimental results are illustrated in Section 4. Concluding remarks are finally given in the last section.

## 2. Structure Design, Simulation and Fabrication

### 2.1. Structure Design

**Figure 1.**
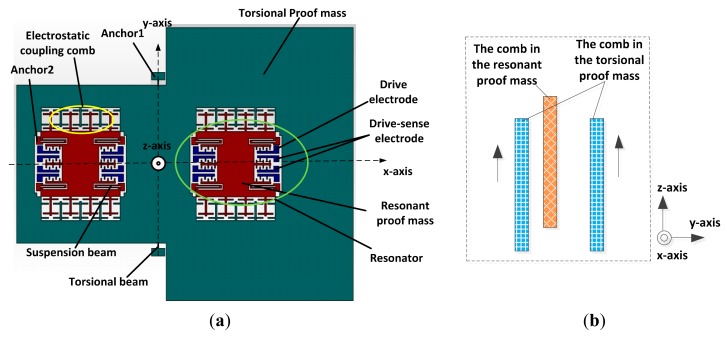
The scheme of a *z*-axis resonant micro-accelerometer. (**a**) The structure of the micro-accelerometer; (**b**) Partial sectional view of the electrostatic coupling comb.

The structure of the new *z*-axis resonant micro-accelerometer is shown in Figure 1A. The *z*-axis resonant micro-accelerometer, including the torsional proof mass, resonant proof mass, torsional beam, suspension beam, drive electrode, drive-sense electrode, electrostatic coupling comb, anchor 1/2, and so on, consists of a torsional accelerometer and two plane resonators. Firstly, the resonant proof masses on both sides of the structure will be driven respectively to vibrate in the natural frequency of the resonators along the *y*-axis. The resonant displacements can be detected by the drive-sense electrodes. By feeding back the resonant displacements to the drive electrodes, the closed-loop self-oscillation system is implemented and can track the natural frequency of the resonator. Secondly, the bias voltage between the torsional proof mass and the resonant proof mass is applied on the electrostatic coupling combs. The electrostatic force and electrostatic stiffness are generated. Finally, when the *z*-axis acceleration is input, due to the imbalance of the torsional proof mass on both sides, the torsional proof mass will be rotated through the torsional beam around the *y*-axis. The overlapping area of the electrostatic coupling combs shown in the Figure 1B will be changed, which will cause the change in the electrostatic force and electrostatic stiffness. Therefore, the natural frequency of the resonator is altered as a result of the change in the electrostatic stiffness. By measuring the frequency changes in the closed-loop self-oscillation system, the acceleration can be detected. The new *z*-axis resonant micro-accelerometer decouples the *z*-axis sensing movement of the accelerometer from the oscillation of the plane resonators by electrostatic stiffness. Therefore, the vertical resonator that is ordinarily used to convert the *z*-axis input accelerometer is averted. The entire accelerometer is essentially a plane structure, and the vertical gap is not necessary to control (the vertical gap must be controlled to within several micrometers in the vertical resonator in order to increase the resonant accelerometer sensitivity; however, due to the influence of “pull-in”, too small of a gap is very difficult to achieve), which has good compatibility with the planar process and is conducive to integrating the vertical resonant accelerometer into the plane resonant accelerometer to implement a triaxial resonant micro-accelerometer [16].

The torsional proof mass, the torsional beam and the anchor constitute the basic torsional accelerometer. The equation of motion is:

(1) $$j\frac{d^{2}\theta \left(t\right)}{dt^{2}}+b\frac{d\theta \left(t\right)}{dt}+k_{o}\theta \left(t\right)=k_{a}a\left(t\right)$$

where *J* is the moment of inertia, *b* is the viscous damping coefficient, *k*_o_ is the torsional stiffness, *k*_a_ is the torque coefficient and *θ* is the angle displacement.

In the static state, the output angel displacement is:

(2) $$\theta \left(t\right)=\frac{k_{a}}{k_{o}}a\left(t\right)$$

**Figure 2.**
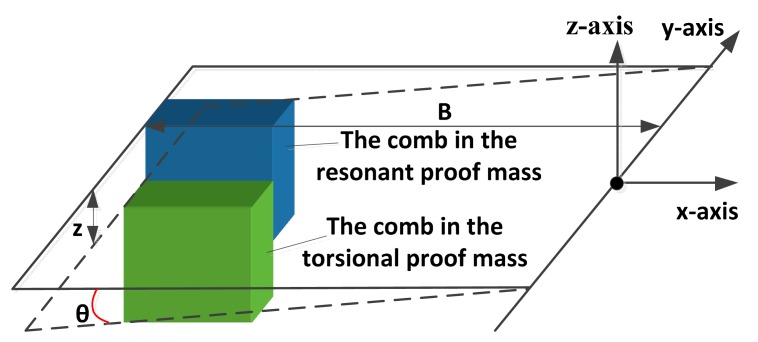
The scheme of the comb movement.

The scheme of comb movement is shown in Figure 2. The displacement of the electrostatic coupling comb along the *z*-axis is:

(3) $$z=B\text{Sin}\left(\theta \left(t\right)\right)\approx B\frac{k_{a}}{k_{o}}a\left(t\right)$$

where *B* is the equivalent distance from the electrostatic coupling combs to the *y*-axis.

The equation of motion of the resonator is:

(4) $$m\frac{d^{2}y\left(t\right)}{dt^{2}}+c\frac{dy\left(t\right)}{dt}+ky\left(t\right)=F_{d}\left(t\right)+F_{e}\left(t\right)$$

where *m* is the resonant proof mass, *c* is the viscous damping coefficient, *k* is the stiffness coefficient of the suspension beam, *F*_d_(*t*) is the drive force and *F*_e_(*t*) is the coupling electrostatic force of electrostatic coupling comb.

**Figure 3.**
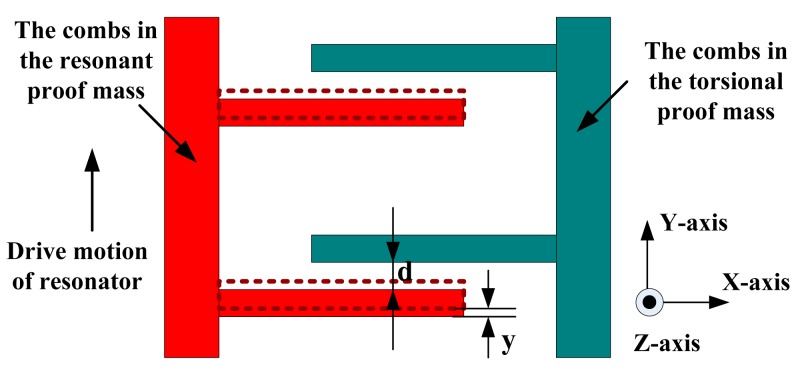
The scheme of electrostatic coupling theory.

The scheme of electrostatic coupling theory is shown in Figure 3. The electrostatic coupling capacitances in the top and the bottom are, respectively:

$$\begin{array}{cc} C_{1}=n\frac{\varepsilon L\left(h+z\right)}{d-y} & C_{2}=n\frac{\varepsilon L\left(h+z\right)}{d+y} \end{array}$$

where *d* is the coupling comb gap, *h* is the comb thickness and *L* is the length. The coupling electrostatic force is:

(5) $$F_{e}\left(t\right)=\frac{1}{2}\frac{\partial C}{\partial y}V^{2}=\frac{1}{2}\frac{n\varepsilon L\left(h+z\right)}{{\left(d-y\right)}^{2}}V^{2}-\frac{1}{2}\frac{n\varepsilon L\left(h+z\right)}{{\left(d+y\right)}^{2}}V^{2}\approx \frac{2n\varepsilon L\left(h+z\right)V^{2}}{d^{3}}y=k_{o}\left(h+z\right)y$$

where *V* is the bias voltage of the electrostatic coupling beam, *k*_e_ = 2*nεLV*^2^/*d*^3^.

Substituting Equation (5) into Equation (4), the natural frequency is:

(6) $$f=\frac{1}{2\pi }\sqrt{\left(k-k_{e}\left(h+z\right)\right)/m}$$

Expanding Equation (6) with the Taylor method:

(7) $$f=f_{0}+{f^{\prime }}_{0}z$$

where *f*_o_ is the static frequency of the resonator and $f_{0}=\left(\sqrt{\left(k-k_{e}h\right)/m}\right)/\left(2\pi \right)$, *f*′_0_ = *nɛLV*^2^/(4*π*^2^*f*_0_*d*^3^*m*).

Substituting Equation (3) into Equation (7):

(8) $$f\approx f_{0}+Sa\left(t\right)$$

where *S* is the scale factor of the single resonator, and *S*=*nɛLV*^2^*Bk_a_*/(4*π*^2^*f*_0_*d*^3^*mk_o_*).

**Figure 4.**
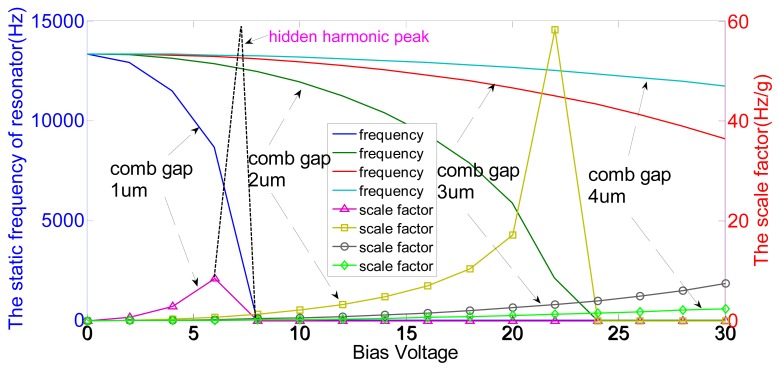
The relationship among the bias voltage, coupling comb gap, the static frequency of resonator and the scale factor (The left is the relationship between the static frequency of resonator *f*_0_ and the bias voltage *V* in the different coupling comb gaps *d*, presented with different colors, blue “ 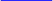
”, dark green “ 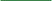
”, red “ 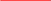
” and light blue “ 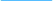
”, at the top of the figure. The right is the relationship between the scale factor S and the bias voltage *V* in the different coupling comb gaps *d*, presented with different symbol of “Δ”, “□”, “○” and “◊” in the bottom of the figure. When the coupling comb gap *d* = 1 μm, a harmonic peak of the scale factor is missed in the bias voltage between 5 V and 10 V, shown in the curve of the symbol “∆”. Therefore, a hidden harmonic peak is added with the black dotted line).

It is evident that the scale factor can be increased by decreasing the *f*_0_, *d*, *m* and *k*_o_ or increasing the *n*, *L*, *V*, *B* and *k*_a_. The design structure parameters are shown in Table 1. Figure 4 shows the relationship among the bias voltage *V*, coupling comb gap *d*, the static frequency of resonator f_0_ and the scale factor *S*. The negative stiffness coefficient of the electrostatic coupling comb and scale factor rise simultaneously with increasing the bias voltage *V*. When *k* = *k*_e_(*h* + *z*), the scale factor has the maximum value and the resonator is operated at critical stability, shown in the hidden harmonic peak and the yellow harmonic peak with the symbol “□” in Figure 4. Theoretically, the comb gap should be reduced to the minimum in order to maximize the scale factor. However, a relatively small comb gap will obviously increase the sensitivity of the scale factor to the bias voltage, especially around the stable point, such as the 1-μm comb gap shown in Figure 4. Considering the sensitivity of the scale factor and the stable interval of the bias voltage synthetically, a 2-μm coupling comb gap is chosen.

### 2.2. Simulation

**Figure 5.**
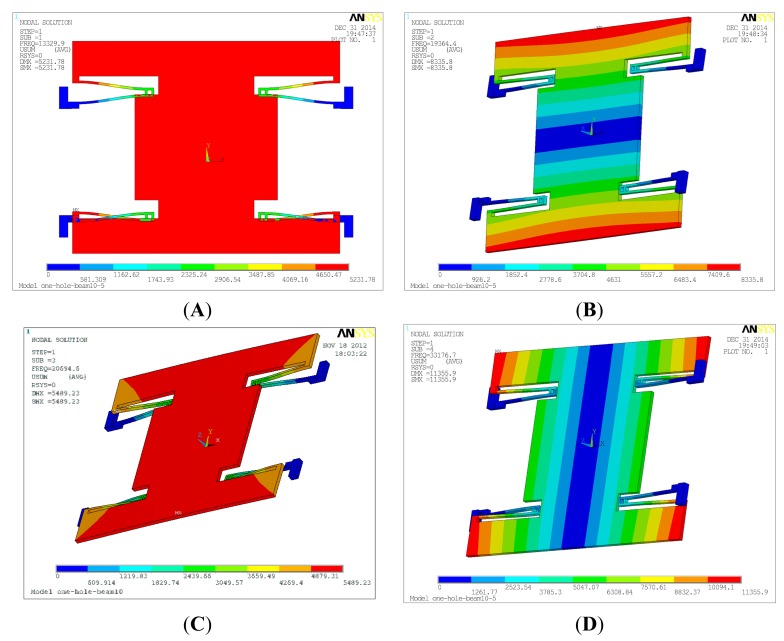
The mode simulation of the resonator. (**A**) The first mode; (**B**) The second mode; (**C**) The third mode; (**D**) The fourth mode.

In order to verify the basic principle of the structure, the simulation is implemented by Ansys. The resonators are coupled with the torsional accelerometer by the electrostatic stiffness. It is difficult to simulate the whole structure directly. Therefore, the resonators and the torsional accelerometer are simulated separately. The mode simulation results of the resonator are shown in Figure 5. The first mode is the effective resonant mode along the *y*-axis, and the frequency is 13.3 kHz. Theoretically, the frequency of the first mode should be selected as small as possible. However, the electrostatic negative stiffness will lead to the decrease of the effective resonant frequency, shown in Figure 4. The suspension beams should be maintained at a certain stiffness in order to ensure the system stability. The other three are the interference modes. The interference modes shown in the Table 2 are apparently isolated with the effective mode. The resonator is driven to vibrate in the first mode. Additionally, the closed-loop self-oscillation system is locked in the first mode.

**Figure 6.**
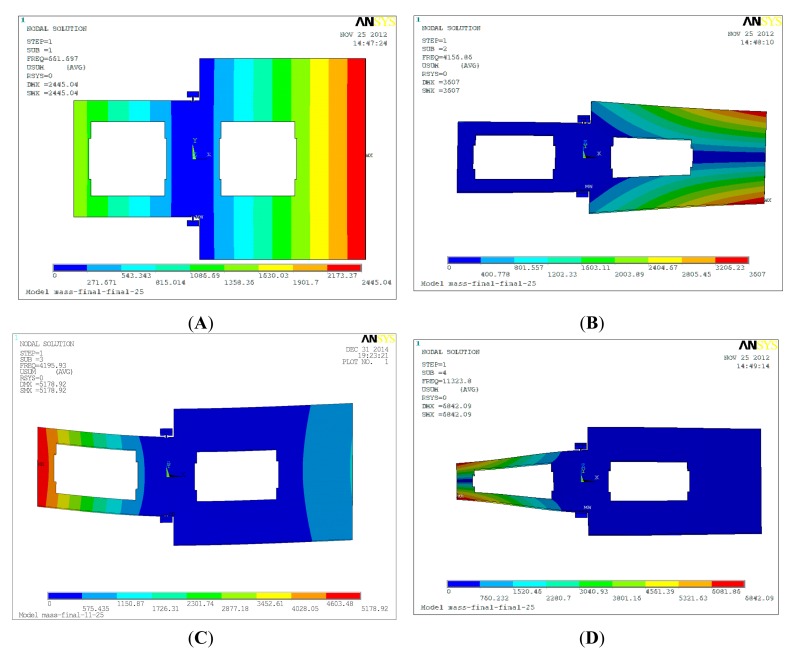
The mode simulation of the torsional accelerometer. (**A**) The first mode; (**B**) The second mode; (**C**) The third mode; (**D**) The fourth mode.

The mode simulation of the torsional accelerometer is shown in Figure 6. The first mode is the effective torsional resonant mode around the *y*-axis, and the frequency is 0.66 kHz. Similarly, the frequency of the first mode should be selected as small as possible in order to increase the sensitivity. However, the lower resonant frequency will also result in a stiffness decrease along the *z*-axis direction, which will cause the asymmetry of displacement in the left and the right. The other three are the interference modes. The interference modes shown in Table 2 are isolated with the effective mode.

### 2.3. Fabrication

**Figure 7.**
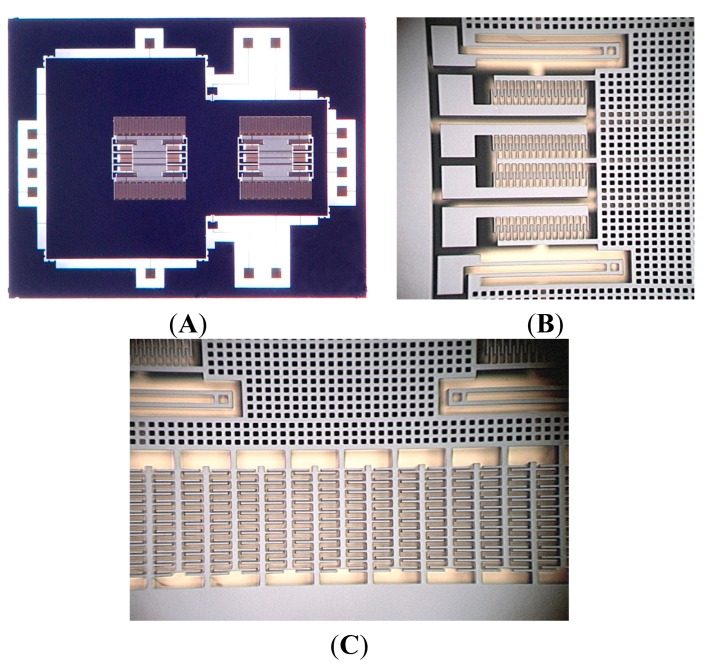
Picture of the fabricated structure. (**A**) The whole mechanical structure; (**B**) Partial view of the resonator structure; (**C**) Partial view of the electrostatic coupling comb.

The new *z*-axis resonant micro-accelerometer has been fabricated by the standard deep dry silicon on glass (DDSOG) process. A single four-inch crystalline silicon wafer is adopted. The process flow consists of: (1) laying photoresist on the silicon wafer and photo etching; (2) Deep reactive ion etching (DRIE) to form bonding area; (3) sputtering of a Cr/Ti/Au layer on 7740 Pyrex glass to fabricate the electrode wire; (4) Si/glass electrostatic bonding; (5) reducing the silicon wafer thickness to 25-μm by KOH wet etching and polishing; and (6) DRIE with 20:1 aspect ratio etching to release the structure. The gap in the electrostatic coupling comb has a measured value of 2.5-um, which is larger than the design value of 2-μm. The larger gap will decrease the scale factor. Figure 7 shows the picture of the fabricated structure. Figure 7A shows the whole structure of 6900 μm × 5300 μm × 20 μm. Figure 7B,C shows the partial view of resonator and the electrostatic coupling comb.

## 3. Design of the Oscillation Loop and the Frequency Measurement Circuit

**Figure 8.**
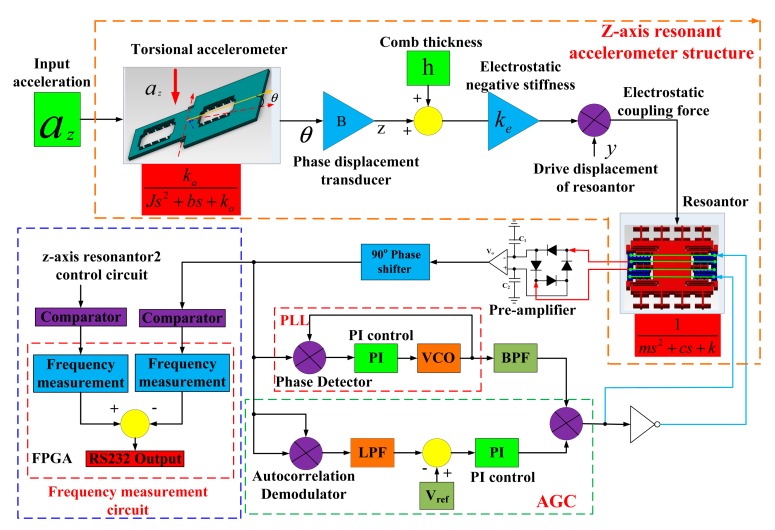
The detecting and control circuit for the *z*-axis resonant accelerometer.

Figure 8 shows the detecting and control circuit for the *z*-axis resonant accelerometer. The detecting and control circuit achieves three functions: the closed-loop self-oscillation, the frequency locking of natural frequency and the frequency measurement. The vibration displacement of the resonator is firstly detected by the pre-amplifier with the ring diode demodulator. Then, the phase is revised by the “90° phase shifter”. Finally, the self-oscillation is realized by the auto gain control (AGC) and the phase locked loop (PLL). The AGC, which is used to control the vibrating amplitude of the resonator at a constant value, includes the autocorrection demodulator, low pass filter (LPF), subtracter, proportional integral (PI) control and the multiplier. Comparing to silicon micro-gyroscope, the resonator has a higher resonant frequency and a smaller vibration displacement. Therefore, the vibration signal is likely affected by the environmental noise. The autocorrection demodulator is utilized to suppress the noise in order to obtain the real vibration amplitude. The PLL, which consists of the phase detector, the PI control and the voltage-controlled oscillator (VCO), cooperates with the “90° phase shifter” to lock the system oscillation frequency to the natural frequency of the resonator. Due to the nonlinearity of the resonator and electrostatic coupling force, the output of pre-amplifier has a harmonic wave. When the output of PLL is designed to lock to the dominant frequency, the phase detector can suppress the influence of harmonic wave and noise. At last, the signal frequency of the oscillation loop is measured by FPGA.

## 4. Experiment

**Figure 9.**
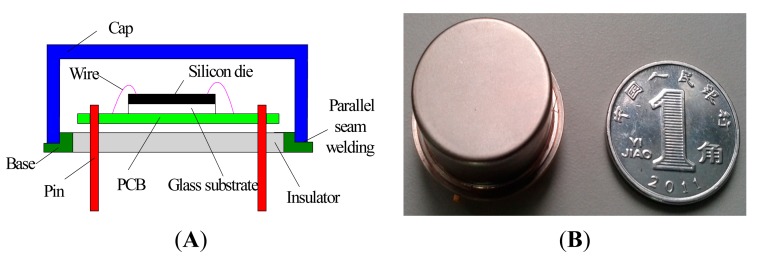
Vacuum encapsulation. (**A**) Schematic diagram of vacuum encapsulation; (**B**) Prototype of vacuum encapsulation.

**Figure 10.**
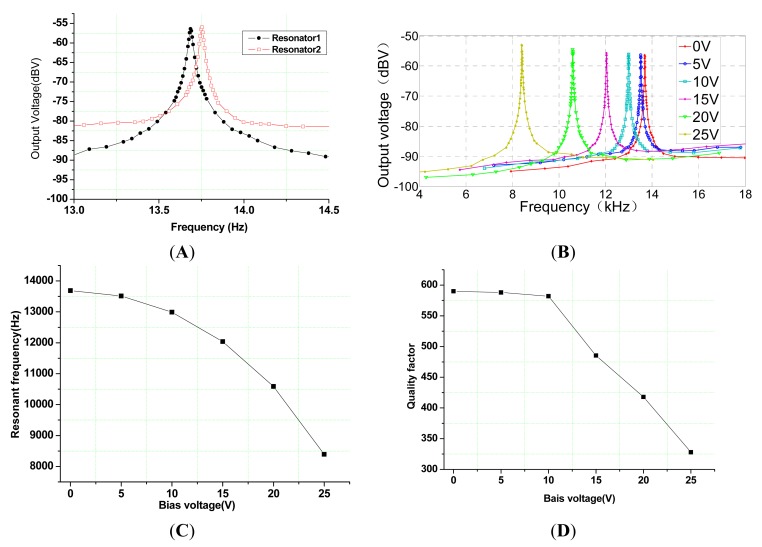
The mechanical features experiment of the *z*-axis resonant micro-accelerometer structure. (**A**) The curve of resonant frequency and quality factor; (**B**) The resonant characteristic curve of Plane Resonator 1 in the different bias voltage applied on the electrostatic coupling comb; (**C**) The relation curve between static resonant frequency and bias voltage applied on the electrostatic coupling comb; (**D**) The relation curve between the quality factor and bias voltage applied on the electrostatic coupling comb.

The experiments are implemented to verify the feasibility of the new *z*-axis resonant micro-accelerometer. In order to decrease the phase noise and improve the closed-loop control performance, the structure chip of the *z*-axis resonant micro-accelerometer is packaged by parallel seam welding in vacuum conditions to improve the quality factor, shown in Figure 9. The mechanical features of the *z*-axis resonant micro-accelerometer structure shown in Figure 10 are measured under a 100-mTorr vacuum degree in the device. The two resonators shown in Figure 10A are measured in the 0-V bias voltage. The resonant frequencies are 13.686 kHz and 13.753 kHz, and the quality factors are 590 and 647, respectively. The quality factor is calculated by the equation f/(f_+_ − f_−_), where f is the resonant frequency, f_+_ and f_−_ present the right and left frequencies of −3 dB attenuation relative to the harmonic peak. The resonant characteristic curve of the Plane Resonator 1 in the different bias voltage applied on the electrostatic coupling comb is shown in Figure 10B. It is evident that the increase of the bias voltage will result in the frequency decrease of the plane resonator. The frequency of the plane resonator will decrease from the 13.686 kHz in the 0-V bias voltage to the 8.392 kHz in the 25-V bias voltage, shown in Figure 10C. The main reason is that the electrostatic force resulting from the electrostatic coupling comb causes the negative stiffness effect and reduces the effective stiffness of Plane Resonator 1, which is consistent with Equation (7). At the same time, the quality factor will decrease with the increase of the bias voltage. The quality factor of Plane Resonator 1 will decrease from 590 in the 0-V bias voltage to 328 in the 25-V bias voltage, shown in Figure 10D. The possible reason is that the electrostatic force increases the structure damping.

**Figure 11.**
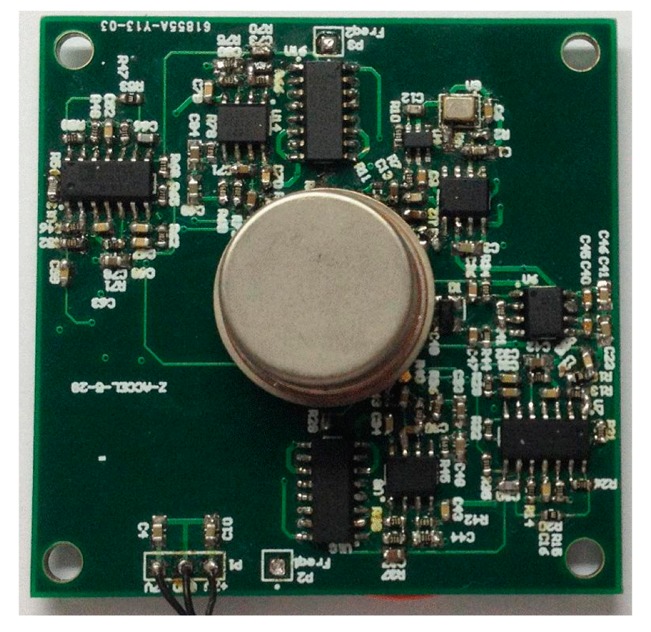
The prototype of the *z*-axis resonant micro-accelerometer.

**Figure 12.**
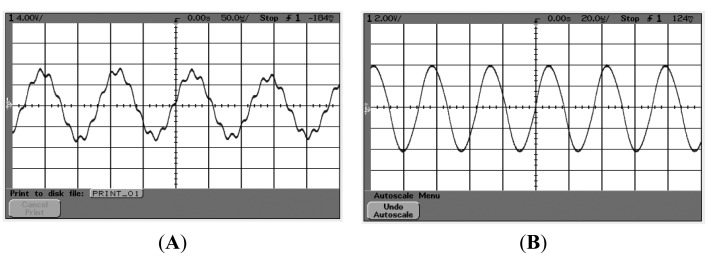
The waveform of harmonic suppression by PLL. (**A**) The waveform before PLL; (**B**) The waveform after PLL and BPF.

**Figure 13.**
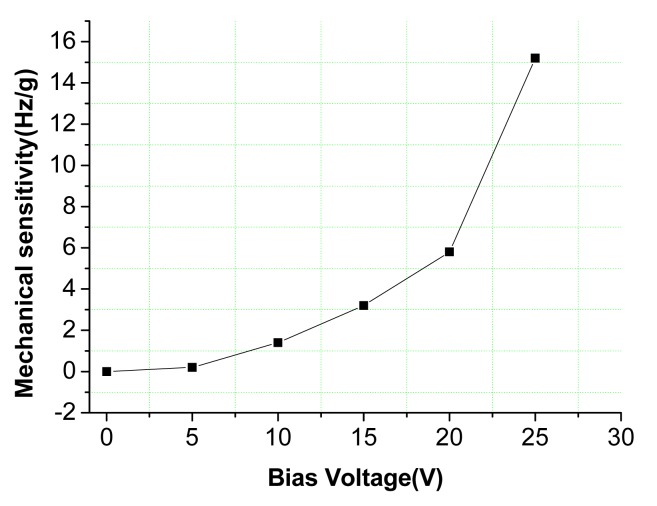
The relation curve between the mechanical sensitivity of the single-plane resonator and the bias voltage applied on the electrostatic coupling comb.

According to the scheme of the oscillation loop and the frequency measurement circuit in Figure 8, the PCB circuit is designed and debugged with the mechanical structure. The prototype of the *z*-axis resonant micro-accelerometer shown in Figure 11 is achieved. The circuit has succeeded in tracking the natural frequency of resonators. The waveform of harmonic suppression by PLL is shown in Figure 12. Due to the nonlinearity of the plane resonator and the influence of the electrostatic force applied on the electrostatic coupling comb, the output of the pre-amplifier has some harmonic signal and noise, shown in Figure 12A. The output of PLL will be locked in the dominant frequency, which will benefit suppression of the harmonic signal and noise, shown in Figure 12B. To confirm the feasibility of the detection principle of acceleration, the mechanical sensitivity of the single-plane resonator is measured in different bias voltages, shown in Figure 13. The mechanical sensitivity only has 15.2 Hz/g in the 25-V bias voltage. The measured mechanical sensitivity is obviously smaller than that of the theory. The main reason is that the fabricated gap is larger than that of the design.

**Figure 14.**
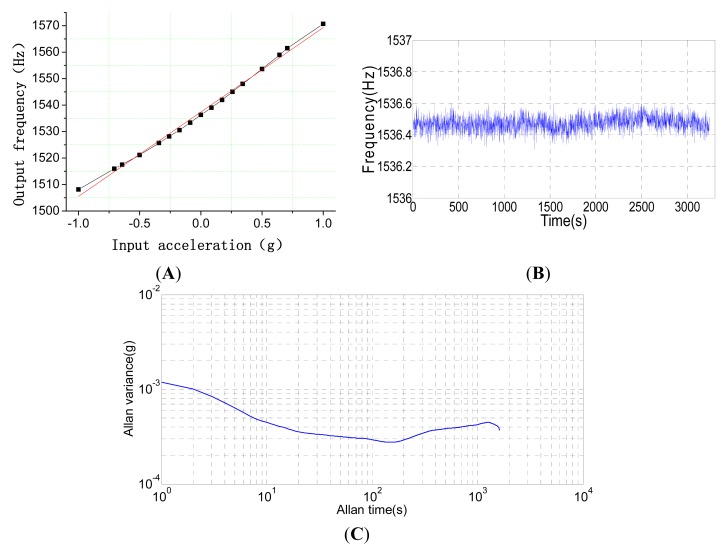
The system performance experiment. (**A**) The curve of output frequency *versus* input acceleration; (**B**) The curve of bias stability of the *z*-axis resonant accelerometer; (**C**) The Allan variance curve of the bias drift of the *z*-axis resonant accelerometer.

The system performance experiment is shown in Figure 14 and Table 3. The system bandwidth is mainly limited by the circuit instead of the structure chip. A multi-cycle synchronous frequency measurement method is adopted to measure the frequency of the resonator in the FPGA circuit, shown in Figure 8. In order to ensure the measurement accuracy of frequency 0.01 Hz, a time window of 0.02 s is set up. In the period of the time window, one counter is used to calculate the number of pulses in the 100 MHz; the other counter is used to calculate the number of cycles of the resonator frequency at the same time. The resonator frequency can be measured by the values of the counters. Therefore, the system circuit bandwidth of 50 Hz is mainly decided by the time window. Additionally, the mechanical bandwidth should be larger than that of the system circuit.

The scale factor experiment was carried out using the goniometer. By changing the angle of the goniometer, the different acceleration rates are input along the input axis. Figure 14A shows the relationship between the input acceleration and the output resonant frequency. The scale factor of the resonant accelerometer along the *z*-axis is 31.65 Hz/g. The frequency sensitivity of the resonant accelerometer has to compromise with the scale factor non-linearity and dynamic range. In order to increase the sensitivity, the torsional beam width of the torsional accelerometer shown in Figure 1 should be decreased. However, too thin of a torsional beam will lead to the *z*-axis translational displacement of the whole torsional proof mass, which will cause the non-linearity of the scale factor. Additionally, the excessive sensitivity will give rise to the large rotational displacement of the whole torsional proof mass and, finally, limit the dynamic range. The cross axis sensitivity of the *x*-axis and *y*-axis shown in Table 3 is measured. Obviously, the cross axis sensitivity of the *x*-axis is larger than that of the *y*-axis. The main reason is that the stiffness of the torsional beam along the *x*-axis is softer than that of the torsional beam along the *y*-axis, shown in Figure 1.

Figure 14B shows the static bias drift of the *z*-axis resonant micro-accelerometer when zero acceleration is applied. The obtained equivalent bias drifts are 727.0 μg with 1σ standard deviation. The Allan variance curve of the bias drifts is shown in Figure 14C. The bias stability of Allan variance is 222.2 μg and the rate of the random walk is $32.6\;\mu \text{g}/\sqrt{s}$. Finally, the performance test results of the *z*-axis resonant micro-accelerometer are summarized in Table 3. The results demonstrate that the principle of *z*-axis resonant micro-accelerometer is feasible.

## 5. Conclusions

The design, simulation, fabrication and experiment of a new *z*-axis resonant accelerometer based on electrostatic stiffness are presented in the paper. The structure of the new *z*-axis resonant accelerometer is illuminated firstly. The sensitive theory of the acceleration is investigated, and the equation of the scale factor is deduced under ideal conditions. The simulation is implemented to verify the basic principle of the torsional accelerometer and the plane resonator individually by Ansys. The structure simulation results prove that the effective frequency of the torsional accelerometer and the plane resonator are 0.66 kHz and 13.3 kHz, respectively. Additionally, the interference modes are apparently isolated with the effective mode. Then, the new structure is fabricated by the standard three-mask DDSOG process and is encapsulated by parallel seam welding. Finally, the detecting and control circuit for the *z*-axis resonant accelerometer is designed to achieve closed-loop self-oscillation, to trace the natural frequency of the resonator and to measure the frequency. Experimental results show that the new *z*-axis resonant accelerometer has a scale factor of 31.65 Hz/g, a bias stability of 727 μg in the *z*-axis and a dynamic range of over 10 g, which proves that the new *z*-axis resonant micro-accelerometer is practicable. Future work includes reducing the nonlinearity and improving the performance.

## Figures and Tables

**Table 1.** The simulation parameters.

**Parameter**	**Value**	**Parameter**	**Value**
*m* (kg)	3.3 × 10^−8^	*V* (V)	18
*c* (N·s/m)	2.8 × 10^−8^	*B* (μm)	1152
*k* (N/m)	232.5	*k*_o_ (N·m/rad)	2.3 × 10^−5^
*n*	242	*k*_a_ (N·m)	7 × 10^−10^
*L* (μm)	35	*J* (kg·m^2^)	3.0 × 10^−12^
*h* (μm)	25	*B* (N·m·s/rad)	2.3 × 10^−8^
*d* (μm)	2	*S* (Hz/g)(theory)	10.43

**Table 2.** The first six modes of the resonator and the torsional accelerometer.

**Mode No.**	**1**	**2**	**3**	**4**	**5**	**6**
Frequency of the resonator (kHz)	13.3	19.4	20.7	33.2	51.6	67.0
Frequency of the torsional accelerometer (kHz)	0.66	4.16	4.20	11.3	11.6	14.9

**Table 3.** The system performance experiment results.

**Test Parameter (Units)**		***z*-axis**
Dynamic range (g)		10
Scale factor (Hz/g)		31.65
Scale factor repeatability (%)		0.85
Scale factor non-linearity (%)		4.49
Scale factor asymmetry(%)		23.29
Zero bias (g)		48.54

Cross axis sensitivity(%)	*x*-axis	5.67

	*y*-axis	1.45

Zero bias stability (μg)	Standard deviation (1σ)	727.0

	Allan variance	222.2

Rate random walk ( $\mu g/\sqrt{s}$)		32.6
Bandwidth (Hz)		50
